# Younger Researchers of the Brazilian Chemical Society: a network fostering representation, communication, and collaboration

**DOI:** 10.1039/d3ra90072j

**Published:** 2023-08-14

**Authors:** Paula C. P. Bueno, Marilia Valli, Eduarda A. Moreira, Helena Mannochio-Russo, Vanessa Nascimento

**Affiliations:** a Leibniz Institute of Vegetable and Ornamental Crops (IGZ) Theodor-Echtermeyer-Weg 1 Großbeeren 14979 Germany bueno@igzev.de paulabueno@yahoo.com; b Federal University of Alfenas (UNIFAL-MG) R. Gabriel Monteiro da Silva 700 Alfenas 37130-001 Brazil; c São Carlos Institute of Physics (IFSC), University of São Paulo (USP) Av. João Dagnone 1100 São Carlos SP 13563-120 Brazil marilia.valli@ifsc.usp.br; d Department of Chemistry, Federal University of São Carlos Washington Luis Highway s/n Km 235 São Carlos SP 13565-905 Brazil em.antunes@yahoo.com.br; e Skaggs School of Pharmacy and Pharmaceutical Sciences, University of California San Diego La Jolla CA 92093 USA hmannochiorusso@health.ucsd.edu; f Department of Organic Chemistry, Institute of Chemistry, Universidade Federal Fluminense Campus do Valonguinho Niterói RJ 24020-141 Brazil nascimentovanessa@id.uff.br

## Abstract

The Younger Researchers of the Brazilian Chemical Society committee supports early career researchers promoting communication, collaboration, education, networking, representation, and career development.
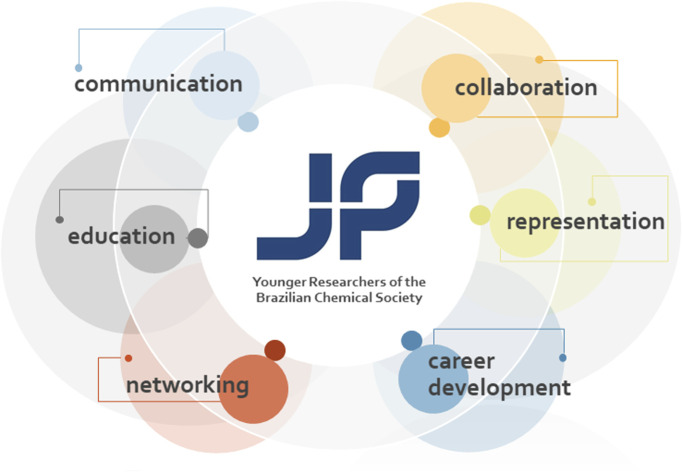

The establishment of national and international scientific networks has been progressively encouraged by scientific and governmental entities worldwide. This movement aims to strengthen science and technology sectors, given the advantageous intersection of ideas, promotion of interdisciplinary projects, and stimulation for creating multilateral opportunities in research and innovation. We live in a constantly changing world, and we must be prepared to handle local and worldwide challenges that emerge from the social, economic, and health fields. In this regard, strategic decisions and sustainable actions depend largely on the exchange of knowledge, flexibility to deal with adversities, and the ability to manage complex situations.

Scientific societies play an essential role in this process by aggregating experts in their areas. However, it is a challenge for their knowledge to be further fructified into the preparation of future leaders and training them as new decision-makers. Indeed, there is a common sense that the active participation of younger generations of researchers in scientific societies is vital for the dissemination of the knowledge acquired by experienced scientists who have been building those societies.^1^ Undoubtedly, the creation and maintenance of networks composed and directed to young researchers are highly beneficial for both the younger and senior scientists. Whereas experienced researchers provide valuable training, mentoring, and professional advice that contribute to the younger researcher’s career advancement, early career researchers provide up-to-date information about new and upcoming technologies in an era of rapid digitalization, for example. Besides, they can also offer their undeniable enthusiasm and fresh ideas.^2^

Bringing the discussion to the Latin American scenario, this can be illustrated by the creation of the committee of Younger Researchers of the Brazilian Chemical Society (JP-SBQ),^3^ which started in 2018. Its first activities included the alignment with the demands of the Brazilian Chemical Society (SBQ) board and divisions, the search for institutional support, writing bylaws, and defining the initial activities for its consolidation. The committee would have its existence centered on the organization of a workshop during the SBQ annual meetings and on the representation in national and international meetings and initiatives. However, after the first months of its launch, the committee took on other roles, both within the Brazilian Chemical Society itself and in the broader scientific community. These activities highlighted the enormous demand already existing in the scientific environment, and the potential of young researchers as agents of transformation, renewal of ideas, and workforce. After this initial structuring, the official launch occurred in 2019 during the 42nd Annual Meeting of the Brazilian Chemical Society (42nd RASBQ, Joinville/SC, Brazil). Since then, the committee has been promoting the representation of Brazilian, young and early-career researchers, in the national and international scientific community. Among several activities and objectives, the committee aims to organize and support the community of younger scientists, fostering communication, collaboration, education, networking, exchange, and career development in an inclusive and effective way. Both in the national and international scenario, the core activities include: (1) full support of several actions headed by the Brazilian Chemical Society; (2) governance; (3) public outreach; (4) social media management; (5) assistance, coordination and transmission of web-based courses, seminars and podcasts; (6) organization of events during the SBQ annual meetings; (7) representation and participation in external conferences; (8) organization of journal themed issues dedicated to young researchers; (9) structuring and launching recognition awards.

The principles of equality, inclusion, freedom of ideas, and a range of views are also fundamental premises of the committee. It supports people from all ethnicities, sexual orientations, gender identities, socioeconomic status, disabilities, and other individualities. The result is the creation of an environment where all young researchers feel represented and comfortable in a safe, respectful, harassment-free, accessible, and welcoming space.

To promote professional collaboration and interaction between young researchers from different chemistry fields from companies or universities, the network includes representatives from most Brazilian regions. However, in a continental country such as Brazil, promoting the development of a network that reaches towards underrepresented regions is not an easy task. Overcoming this bottleneck by increasing diversity, exchange of ideas, and, finally knowledge advancement, is the aim.

Another key aspect of the committee’s activities involves career development in a country threatened by unstable funding for science, inadequate financial support, and PhD salaries based on monthly scholarships without any professional register, such as retirement or payroll taxes contribution.^4^ The committee advocates for urgent revision of the governmental policies, turning them into enduring state policies for investments in science, technology, and innovation. This agenda could also be promoted by the mobilization of society, industrial, and academic bodies to put Brazil on a path to becoming a developed and self-sufficient country and not an exporter of the young science talent. By these means, proper investment in highly skilled early-career scientists will contribute to building a modern and sustainable economy based on human capital, technology, and innovation, rather than mainly on the exploitation of natural resources.^5^

Implementing a committee composed of young researchers in the Brazilian Chemical Society was only possible due to the support of senior and well-established researchers, as well as the support of university bodies, research, and funding institutions. In common, those bodies are aware that investments in young leadership are essential to address national and global challenges.^6^

Prof. Dr Shirley Nakagaki, current president of SBQ, says:

“*SBQ is the largest scientific society for Chemistry in Brazil. It has a long history of representing chemists in all instances where such representation is necessary, whether in academia, research funding agencies or committees involved in formulating and making decisions about current scientific and technological policies in the country. For us, the board of the Brazilian Chemical Society, it has always been a challenge to involve our young researchers in the society’s activities, as we understand that without active young researchers and decision-makers, no scientific society has a future. Investing in our young scientists, equipping them with the skills they deem important, is key to building a sustainable society. Guiding them along the scientific path, igniting their interest in science, particularly in the field of chemistry, begins in academia, research laboratories, and the development of scientific work that, in most cases, is destined for success. We, as educators and researchers, are trained for this. However, showing them the path involved in making the right decisions regarding their involvement with universities and representation committees that guide research policies, is a fundamental role in establishing a solid and fruitful scientific community in our country. In scientific organizations like SBQ, we have groups of individuals with this expertise, and the creation of committees like JP-SBQ serves as an open and efficient channel to provide our young researchers with the necessary information and training to achieve this learning in an appropriate, ethical, and scientific environment. As the current president of SBQ, and undoubtedly speaking on behalf of my predecessors, I have been striving to accommodate the demands and ideas of our young researchers in order to make SBQ a facilitating environment for their lifelong scientific learning. However, within the society, we certainly possess the expertise to facilitate this knowledge acquisition. Our goal, from the perspective of JP-SBQ, is to have our young researchers become national and international scientific leaders, trusted educators, influencers, and decision-makers who will help shape Brazilian scientific policies.*”

## Supplementary Material
